# Molecular pathogenesis of *Chlamydia trachomatis*

**DOI:** 10.3389/fcimb.2023.1281823

**Published:** 2023-10-18

**Authors:** Brittany Jury, Charlotte Fleming, Wilhelmina M. Huston, Laurence Don Wai Luu

**Affiliations:** ^1^ School of Life Sciences, Faculty of Science, University of Technology Sydney, Ultimo, NSW, Australia; ^2^ Faculty of Science, University of Technology Sydney, Ultimo, NSW, Australia

**Keywords:** *Chlamydia trachomatis*, pathogenesis, virulence, immune evasion, type III secretion system, proteases, cytotoxin, polymorphic membrane proteins

## Abstract

*Chlamydia trachomatis* is a strict intracellular human pathogen. It is the main bacterial cause of sexually transmitted infections and the etiologic agent of trachoma, which is the leading cause of preventable blindness. Despite over 100 years since *C. trachomatis* was first identified, there is still no vaccine. However in recent years, the advancement of genetic manipulation approaches for *C. trachomatis* has increased our understanding of the molecular pathogenesis of *C. trachomatis* and progress towards a vaccine. In this mini-review, we aimed to outline the factors related to the developmental cycle phase and specific pathogenesis activity of *C. trachomatis* in order to focus priorities for future genetic approaches. We highlight the factors known to be critical for developmental cycle stages, gene expression regulatory factors, type III secretion system and their effectors, and individual virulence factors with known impacts.

## 1 Introduction

The sexually transmitted pathogen *Chlamydia trachomatis* (CT) is the leading cause of preventable blindness worldwide (Taylor et al., 2014) and the most common bacterial sexually transmitted infection (STIs) in humans, with approximately 131 million cases each year and rising (Newman et al., 2015). The majority of chlamydial infections are asymptomatic. The infection when diagnosed can be treated with antibiotics (Scidmore, 2009). Complications of CT infections are known to include reproductive tract impacts which can cause considerable morbidity and cost (Davies et al., 2016).

The *Chlamydiae* are a ubiquitous family of Gram-negative pathogens, with a unique lifecycle. *Chlamydiae* species causes infection in specific animals, such as *Chlamydia muridarum* in mice and hamsters (Mishkin et al., 2022), while *Chlamydia psittaci* commonly infects birds and rarely humans (Hogerwerf et al., 2020). *Chlamydia pneumoniae* and CT are endemic to humans, they infect different anatomical sites, respiratory and oculogenital, respectively (Whittum-Hudson and Hudson, 2005). CT is further divided into serovars based on the hypervariable region of the major outer membrane protein (MOMP) (Hepler et al., 2018). CT serovars exhibit different tissue tropisms. A-C infects ocular tissue and causes trachoma. D-K primarily infects the genital tract resulting in the STI ‘chlamydia’ but in newborns can also infect respiratory and ocular conjunctiva tissues causing pneumonia and conjunctivitis. L1-L3 infects genital tissues and macrophages resulting in the invasive STI ‘lymphogranuloma venereum (LGV)’ (Abdelsamed et al., 2013).

**Figure 1:**
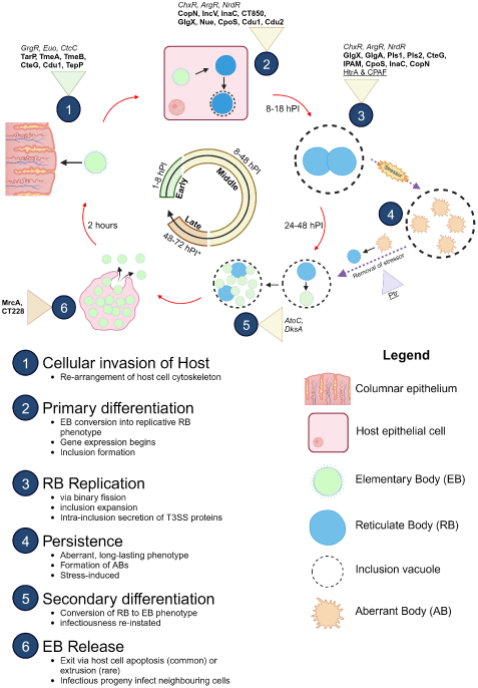
Developmental cycle of *C. trachomatis*. Important proteins expressed at different stages of the development cycle are shown with established transcription factors (italicised), T3SS effectors (bolded), and proteases (underlined). Infectious EBs (coloured green) adhere to and enter the host cell (coloured pink) using various adhesins (Ctad1, Pmps) and the T3SS. Once inside, EBs begin differentiation into replicative RBs (coloured blue). During the RB replication phase, host cell metabolites are used to facilitate the pathogen’s survival. After several rounds of replication, the RBs then asynchronously re-differentiate into EBs for exit via extrusion of the inclusion vacuole from the host cell or lysis of the host cell. The released EB progeny then restarts the infection cycle. Release of EBs (late stage) varies from 48 hours to 72 hours, depending on the serovar (asterisk). In atypical cycles, stressors trigger persistence, in which RBs (coloured green) pause replication and differentiate into long-surviving, stable ABs (coloured orange) and enter a stasis-like state until the stressor is removed. The ABs “detect” this change and re-differentiate back into replicative RBs to resume development. Figure produced with BioRender.

The *Chlamydia* developmental cycle is characterised as biphasic, relying on specific interactions with the host cell for nutrients in order to survive and replicate due to the reduced chlamydial genome (Gitsels et al., 2019). There are two main morphological bacterial phases; the extracellular infectious elementary body (EB) and the intracellular, non-infectious, replicative reticulate body (RB) [reviewed in (Hafner et al., 2008; Elwell et al., 2016; Witkin et al., 2017)] (**Figure 1**). The infectious EBs are robust formations with spore-like structures that consist of disulphide cross-linked outer membrane protein complex on the surface. The development cycle of CT can be impacted by environmental factors and stresses. Known stressors include nutrient deprivation, exposure to host cytokines, and cell wall synthesis-targeting antibiotics (Gitsels et al., 2019). Under these conditions, RBs transition to altered morphological forms of non-culturable, non-dividing, aberrantly enlarged “persistent” forms, called aberrant bodies (ABs) (Panzetta et al., 2018). These forms remain viable and can revert into an active infection once conditions are favourable (Schoborg, 2011).

As an obligate intracellular organism, virulence and pathogenic features interplay critically with host cellular factors and the host immune system which can also contribute to disease pathology [reviewed in (Geisler, 2010; Menon et al., 2015; Murthy et al., 2018; Chen et al., 2019)]. The intracellular nature and unusual bi-phasic developmental cycle has, until recently, hampered progress on biological investigations and genetic manipulation of *Chlamydia*. Recently, genetic methods have rapidly progressed and accordingly our understanding of pathogenic mechanisms has increased (Fisher and Beare, 2023). However, each genetic approach has their advantages and limitations [reviewed in, (Banerjee and Nelson, 2021; Wan et al., 2023)]. Previously, *Chlamydia* was thought to be genetically intractable due to its intracellular development, vacuole niche, evasive persistence mechanism, serovar genetic variability, and the thick wall of EB absorbing external DNA (Andersen et al., 2021). As such, specialised techniques were developed to better understand this clinically significant pathogen. Whole genome sequencing (Stephens et al., 1998), allelic exchange mutagenesis (Mueller et al., 2016), CRISPRi knockdown (Ouellette, 2018; Ouellette et al., 2021) and intron insertion gene inactivation (O'Neill et al., 2021) assist in associating genes with chlamydial phenotypic traits, bypassing impractical traditional approaches in genome studies [reviewed in (Read and Massey, 2014; Fisher and Beare, 2023; Luu et al., 2023)]. Nonetheless, many of these genetic manipulation approaches for *Chlamydia* are still relatively time consuming and requires expert culture skills, hence we set out to review what is known about potential virulence factors to guide priorities for future genetic experiments. We do not cover metabolic factors, although it is arguable that these are related to pathogenesis for an obligate intracellular pathogen. We also do not cover unknown hypothetical proteins.

## 2 Transcription and regulatory factors as a target to understand pathogenesis

The bi-phasic development is regulated by transcription factors resulting in distinct gene expression profiles characterised as early, mid-, or late response. *Chlamydia*’s genome encodes a small number of known transcription factors (outlined in **Table 1A**) and sigma factors (σ) (Akers et al., 2011). The trio of chlamydial sigma factors (σ^66^, σ^28^, and σ^54^) serve as RNA polymerases to various promotor genes (Nicholson et al., 2003; Wurihan et al., 2021). Belland et al. (2003) detected σ^28^ and σ^54^ mRNA mid-development at 8 h PI (hours post infection), whilst σ^66^ was detected earliest at 3 h PI. σ^66^ is responsible for activating the majority of CT promoters, whilst select late genes rely on σ^28^- or σ^54^-dependent promoters (Mathews et al., 1999). These sigma factors are regulated by specific transcription factors (**Table 1A**) and anti-sigma factors. A comprehensive review of transcriptional regulation is available from Nicholson et al. (2003) and Belland et al. (2003).

**Table 1A:** *C. trachomatis* transcription factors and their function.

Transcription Factor	Activation in Developmental Cycle	Function	References
Euo	Early – immediately post-entry of EB	Repressor of late gene expression for differentiation into EB during early stages.	(Li et al., 1998; Wurihan et al., 2021)
GrgA	Early	Activates transcription for *euo* and *hcrA* through interaction with σ^66^ and σ^28^ promoters.	(Bao et al., 2012; Wurihan et al., 2021)
HrcA	Late	Conserved regulator of late gene expression of molecular chaperone proteins (e.g. *dnaK* and *groE* operons).	(Wilson and Tan, 2002; Nicholson et al., 2003; Wurihan et al., 2021)
TrpR	Throughout	Negative regulator of tryptophan synthetase (*trpBA*) gene expression.	(Akers and Tan, 2006; Carlson et al., 2006)
ChxR	Mid-cycle	Conserved regulator of signal transduction responses.	(Koo et al., 2006; Barta et al., 2014; Yang et al., 2017)
ArgR (Arginine-dependent gene regulator R)	Mid- to late-cycle	Regulates bacterial arginine biosynthesis and transport.	(Schaumburg and Tan, 2006)
NrdR	Mid- to late-cycle	Regulates ribonuclease reductase transcription, dependent on intracellular nucleotide levels.	(Kolberg et al., 2004; Case et al., 2011)
CtcC (AtoC)	Late (transition from RB to EB)	Predicted σ^54^ RNA polymerase holoenzyme activator to regulate phenotype differentiation and *hrcA*.	(Koo and Stephens, 2003; Soules et al., 2020; Huang et al., 2022)
YtgR	Throughout	Iron-dependent transcriptional repressor regulator (*ytg* operon) and tryptophan salvage pathway.	(Akers et al., 2011; Thompson et al., 2012; Pokorzynski et al., 2019)
DksA	Late (transition from RB to EB)	Transcription “late” gene regulator responding to environmental “stress” stimuli.	(Mandel et al., 2022)

To date, only two anti-sigma factors which inhibit σ^66^ activity have been characterised: CT663, a T3SS Scc4 chaperone, and the switch-protein Regulator of Sigma B W (RsbW) kinase (Rao et al., 2009) (Thompson et al., 2015). RsbW forms a regulatory network with its own anti-anti-sigma factor RsbV1 and the phosphatase RsbU. The activity of RsbW is driven by the levels unphosphorylated RsbV1, where a deficit of RsbV1 drives the switch-kinase towards antagonizing σ^66^, altering transcription of σ^66^ development-related genes involved in metabolism and growth (Hanson et al., 2015; Thompson et al., 2015).

Recently, transcriptional profiles for the σ^54^ regulon was generated using CtcC (an ATP-hydrolyzing response regulator for σ^54^) mutants which further revealed the role of this sigma factor in membrane remodelling and incorporating T3SS effectors into EBs during RB differentiation into infectious EB progeny (Soules et al., 2020). These observations were supported by recent findings from Hatch and Ouellette (2023) which employed CRISPR interference (CRISPRi) gene knockdown of σ^54^. Hatch and Ouellette (2023) also used CRISPRi to knockdown σ^28^ and identified this sigma-factor to be epistatic of σ^54^ and also involved in secondary RB differentiation.

The use of CRISPRi to knockdown and investigate sigma factors has provided new insights into how RB to EB differentiation is regulated (Swoboda et al., 2023). This approach should be adopted to investigate other transcriptional factors in CT (listed in **Table 1A**). It is likely that many regulators will be essential and cannot be inactivated, thus utilising CRISPRi to knockdown will clarify how critical their role is in the CT development cycle as well as determine their regulon.

## 3 Type III secretion system and effector proteins

A well-known pathogenic feature of *C. trachomatis* is the T3SS and their associated effector proteins (Bugalhão and Mota, 2019). The T3SS is composed of a needle-like injectosome apparatus, which secretes effector proteins directly from the chlamydial cytoplasm into the host cell. Specific chlamydial chaperone 4 (Scc4; formerly CT663) precisely regulates T3SS function through gene expression and effector networks (Gao et al., 2020). There are 80 speculated anti-host T3SS-secreted effectors (Betts-Hampikian and Fields, 2010).

The secreted effectors have a variety of functions, including; disrupting the cytoskeletal structure, evasion of host defences, and prevention of host cell apoptosis, described in **Table 1B** (Coburn et al., 2007). Evidence shows the T3SS not only translocates bacterial-encoded proteins into the eukaryotic host cell’s cytoplasm, but also into the inclusion’s lumen (da Cunha et al., 2017). Some effectors (e.g. TarP and TepP) confer protection against cellular immune response triggers, TLR2, NOD1, and STING (stimulator of interferon genes) (Murray and McKay, 2021). The stimulation of IFN-γ from STING results in activation of indoleamine 2,3-dioxygenase (IDO), an integral element of host defence by reducing tryptophan available for the organism. Tryptophan is crucial to CT pathogenesis and genital CT serovars are able to synthesise tryptophan if indole is present through a functional tryptophan synthase (trypA) gene while ocular strains cannot as *trpA* is inactivated (McClarty et al., 2006).

**Table 1B:** Characterised CT T3SS effector proteins and their functions in chlamydial pathogenesis.

Effector Protein	Family Conserved?	Localisation	Host Target/s	Function	References
IncV (CT005)	Specific to *C. trachomatis*	Inclusion membrane	VAPA/B	Tethers the inclusion to the endoplasmic reticulum’s membrane contact sites.	(Stanhope et al., 2017; Stanhope and Derré, 2018; Andersen et al., 2021)
GlgX (CT042)	Specific to *C. trachomatis*	Inclusion lumen	Unknown	Debranches host glycogen.	(Gehre et al., 2016; LaBrie et al., 2019)
Pls1 (CT049)*	Specific to *C. trachomatis*	Inclusion lumen and membrane	Unknown	Proposed to mediate expansion of the inclusion.	(Jorgensen and Valdivia, 2008)
MrcA (CT101)	Specific to *C. trachomatis*	Inclusion membrane	Inositol 1,4,5-triphosphate receptor 3 (ITPR3)	Regulates CT’s release by mediating the myosin phosphatase pathway.	(Nguyen et al., 2018; Sah and Lutter, 2020)
CteG (CT105)	Specific to *C. trachomatis*, *C. muridarum*, and *C. suis*	Golgi complex and plasma membrane of host eukaryotic cell	Unknown	Implicated in eukaryotic cell’s vesicle trafficking and centrosome amplification.	(Pais et al., 2019; Steiert et al., 2023)
IncA (CT119)	Specific to *C. trachomatis*	Inclusion membrane	Vamp 3/7/8	Increases growth rate and production of EBs during replication through homotypic inclusion fusion.	(Suchland et al., 2000; Dehoux et al., 2011; Mueller et al., 2014; da Cunha et al., 2017; Cingolani et al., 2019)
IPAM (CT223)	Specific to *C. trachomatis*	Inclusion membrane	Centrosomal protein 170 (CEP170)	Maintains cytoskeletal stabilisation for vesicular trafficking.	(Dumoux et al., 2015; Olson-Wood et al., 2021)
CpoS (CT229)	Specific to *C. trachomatis, C. caviae*, and *C. muridarum*	Inclusion membrane	Myosin phosphatase	Regulates host vesicle trafficking across the plasma membrane and promotes inclusion growth.	(Rzomp et al., 2006; Andersen et al., 2021)
TarP (CT456)	Yes	Eukaryotic cell cytosol	Actin**, FAK, Vinculin, Rac guanine exchange factors (Sos1, Vav2)	Facilitates invasion of host epithelia.	(Clifton et al., 2004; Clifton et al., 2005; Lane et al., 2008; Mueller et al., 2014; Thwaites et al., 2015; Faris et al., 2020; Pedrosa et al., 2020)
TmeA (CT694)	Specific to *C. trachomatis* and *C. muridarum*	Inclusion membrane and eukaryotic cell plasma membrane	N-WASP GTPase, Ahnak	Facilitates invasion of host epithelia.	(Bullock et al., 2012; Mueller et al., 2014; McKuen et al., 2017; Faris et al., 2020)
TmeB (CT695)	Specific to *C. trachomatis*	Inclusion and eukaryotic cell plasma membrane	Unknown	Implicated function in assisting pathogen uptake into host cell.	(Mueller and Fields, 2015; McKuen et al., 2017; Andersen et al., 2021; Scanlon et al., 2023)
Nue (CT737)	Specific to *C. trachomatis*	Eukaryotic cell nucleus	H2B, H3 and H4	Targets and translocates the host cell nucleus.	(Pennini et al., 2010; Andersen et al., 2021)
InaC (CT813)	Specific to *C. trachomatis* and *C. muridarum*	Inclusion membrane	14-3-3 proteins, ARF1, ARF4, Vamp7 and Vamp8	Regulates inclusion structure and stability and nutrient acquisition.	(Wesolowski et al., 2017; Andersen et al., 2021; Olson-Wood et al., 2021)
CT850	Yes	Inclusion membrane	DYNLT1	Promote inclusion tethering to host cell nucleus.	(Mital et al., 2015)
TepP (CT875)	Specific to *C. trachomatis*	Eukaryotic cell cytosol near the inclusion	CRK, CRKL, GSK3B, PI3K	Prevents neutrophil-mediated recruitment of immune cells and permits prolonged infections.	(Yi-Shan et al., 2014; Andersen et al., 2021; Dolat and Valdivia, 2021)
CopN (Chlamydial outer protein N) (CT089)	Specific to *C. trachomatis* and *C. pneumoniae*	Inclusion membrane	αβ-tubulin	Regulates T3SS effectors secretion and translocation proteins.	(Archuleta et al., 2011; Hanson et al., 2015)

*Currently considered to be a CT T3SS substrate but yet to be experimentally validated.

**TarP direct polymerisation of actin has not been directly associated to plasma membrane reorganisation during EB invasion.

Currently, many proposed effectors remain unconfirmed and even more remain uncharacterised, with the only secretion evidence provided using heterologous T3SS expression systems. By utilising knockout genetics to disable the T3SS, followed by phenotypic and proteomic comparison of wildtype and T3SS-null strains will be important in confirming established and discovering new effectors as well as unravelling their functions.

### 3.1 Tarp

The extracellular translocated actin-recruiting phosphoprotein (TarP, CT456) is required in the early developmental stages (**Table 1B**) (Clifton et al., 2004). TarP is Slc-1-dependent and involved in chlamydial internalisation, and invasion of host cell (Mueller et al., 2014; Faris et al., 2020). TarP binds to the host cell’s major cytoskeletal component, actin. Phosphorylated TarP interacts with a multitude of host cell signalling molecules, as well as phosphatidylinositol 3-kinase (PI3K) and SHC-transforming protein 1 (SHC1), involved in diverse cell functions (e.g. growth, differentiation, motility, survival) (Shehat et al., 2021). TarP was also discovered to impair the highly conserved Hippo signalling pathway in infected epithelial cells, a previously unknown chlamydia-affected pathway involved in cell proliferation and death (Shehat et al., 2021).

### 3.2 TepP

The early translocator phosphoprotein, TepP, is the second Slc-1-dependent effector and one of the few T3SS effectors where null mutants have been characterised (Chen et al., 2014). TepP is secreted by the T3SS to enhance CT infectivity and dampen host immune activation. TepP is localised near the inclusion in the eukaryotic cell’s cytosol, where it is phosphorylated by the host kinases (Carpenter et al., 2017). It then targets host factors CRK, PI3K and GSK3B to locally synthesise phosphoinositide-(3,4,5)-triphosphate (PIP3) and modulate host cell signalling in nascent infections. Specifically, PI3K is linked to inhibiting IFN-induced gene transcription within early inclusions. A TepP null-mutant displayed defected growth and altered host IFN-associated gene expression required to activate early immune responses (Chen et al., 2014). Recruitment of PI3K also suggests a role for TepP in modulating membrane vesicle trafficking events (Carpenter et al., 2017).

### 3.3 Inc proteins

Inc proteins or inclusion membrane proteins, share bilobed hydrophobic domains enabling anchoring to the inclusion membrane. Some Incs contain the presence of vesicle-targeting SNARE-like motifs in their coding sequence (e.g. IncA), which may facilitate host-pathogen interactions (Cingolani et al., 2019; Paumet et al., 2009).CT genome is predicted to encode 55 putative Inc proteins, with 37 confirmed (Weber et al., 2016). There is divergent Inc content across the *Chlamydiaceae* family, each possessing a distinct distribution pattern along the chlamydial inclusion membrane (Mital et al., 2013). IncA is required for inclusion vacuole fusion (**Table 1B**), other functions of Inc proteins include; a possible role in maintaining host cell viability (IncG) (Scidmore and Hackstadt, 2001; Verbeke et al., 2006) and as an early Inc, IncD may be important for establishing the inclusion as a replicative niche (Shaw et al., 2000), although further studies are required to confirm this. Chlamydial T3SS-secreted inclusion factors have been studied in greater detail by da Cunha et al. (2017), and Dehoux et al. (2011). Here, we focus on IncM, CTL0390 and CpoS which have been recently characterised.

The IncM effector targets host cell microtubules, as exhibited by its action in host cell multinucleation, centrosome positioning and Golgi distribution. This was found to impact inclusion morphology stabilisation, as IncM-null mutants exhibited defects in these areas, compared to wildtype strains (Luís et al., 2023).

The CTL0390 Inc effector is thought to play a role in mediating EB exit via host-cell lysis during the late stages. A CTL0390 mutant was observed to have reduced Golgi translocation of STING, which is likely required to regulate host-cell lysis for bacterial exit (Bishop and Derré, 2022).

Lastly, CpoS (chlamydial promoter of survival) suppresses apoptosis and necrosis of host cells to prolong chlamydial infection and increase infectivity (Sixt et al., 2017). CpoS affords protection from within the inclusion, thus can only counteract nearby pro-death host signalling (e.g. STING). CpoS-null mutants exhibited attenuated infections, with expedited clearance from murine genital tract models and reduce propagation in cell cultures. Recently, CpoS was found to block Rab GTPases required for lipid transport regulation, as well as the formation of inclusion microdomains of many Incs needed for pathogen-host cell interactions (i.e. IPAM, IncD and CT222) (Meier et al., 2023).

### 3.4 ChlaDUB

CT868 (ChlaDUB1/Cdu1) and CT867 (ChlaDUB2/Cdu2) are both deubiquitinases which are delivered to the host cytosol, but have differing impacts on CT infectivity. ChlaDUB1 is solely localised to the inclusion membrane where it deubiquinates the anti-apoptosis protein MCL-1. This prevents MCL-1 depletion and apoptosis, thus inhibiting premature host-cell death to allow EB progeny release and increasing infectivity (Fischer et al., 2017). ChlaDUB1 also prevents degradation of host glucose-transporter-1 (GLUT-1) proteins (Wang et al., 2017). This likely supports growth as knockdown of host GLUT-1 impairs *C. trachomatis* infection (Wang et al., 2017). Inactivation of ChlaDUB1 in CT also enhanced sensitivity to IFN-γ and reduced infectivity in murine infections (Fischer et al., 2017). Alternatively, inactivation of ChlaDUB2, which localises at the inclusion membrane and host cytosol, did not impair infection (Pruneda et al., 2018). However, both ChlaDUBs were found to be involved in Golgi fragmentation (Pruneda et al., 2018).

## 4 Adhesins

### 4.1 Ctad1

*C. trachomatis* adhesin 1 (Ctad1) is a highly conserved 47 kDa invasin encoded by CT017. It contains two SH3 (src Homology-3) domains located near the N-terminus. These domains are responsible for Ctad1 binding to integrin β1 subunits on human epithelial cells (Stallmann and Hegemann, 2016). Ctad1 is expressed on the surface of EBs and mediates *C. trachomatis* invasion. Upon binding to integrin β1, Ctad1 triggers activation of the host mitogen-activated protein kinase (MAPK) pathway leading to extracellular signal-regulated kinase 1/2 (ERK1/2) phosphorylation and internalisation of EBs (Stallmann and Hegemann, 2016). Blocking of Ctad1 receptors with recombinant Ctad1 was shown to significantly reduce EB adhesion and internalisation, illustrating its role in virulence (Stallmann and Hegemann, 2016).

### 4.2 PMPs

Polymorphic membrane proteins (Pmps) are a group of type V autotransporter proteins unique to *Chlamydiaceae*, with variable numbers of Pmps encoded on the genomes. In CT, there are 9 Pmps and all nine are involved in adhesion (Becker and Hegemann, 2014).

Pmps consist of a signal peptide, an N-terminus passenger domain and a C-terminus β channel domain. The C-terminus domain allows translocation of the passenger domain through the outer membrane. After translocation through the outer membrane, Pmps can remain surface bound or be further cleaved and secreted into the inclusion. It has been suggested that the different cleavage of PmpD may lead to fragments with different functions (Swanson et al., 2009).

The expression of Pmps are temporally and developmentally driven. For instance, *pmpA* and *pmpI* expression peaked earliest at 12–18 hours post infection and were only detected in RBs (Nunes et al., 2007; Carrasco et al., 2011; Saka et al., 2011). This suggests that both proteins play an important role in RB development. In contrast, *pmpD* peaks mid-cycle at 12-24 hours post infection, whereas expression of other *pmps* (*pmpB-H*) occurred later (Kiselev et al., 2009). This indicates the latter’s involvement in the late stages (such as RB to EB transition) or early stages of infection (EB attachment) (Kiselev et al., 2009; Carrasco et al., 2011; Saka et al., 2011). Remarkably, the expression of the same *pmp* can vary drastically between different strains (Tan et al., 2009) and even in different inclusions for the same culture (Tan et al., 2010). Besides *pmpG*, the regulatory mechanism behind Pmp variation is unknown. The flexibility to alter Pmps expression may allow CT to evade antibody recognition targeted against a specific Pmp.

The importance of Pmps as adhesins in CT pathogenesis is best characterised in PmpD. Pre-incubation with recombinant PmpD was found to inhibit CT attachment (Becker and Hegemann, 2014). CT PmpD null mutants also had a 70% reduced host cell attachment and decreased RBs attached to the inclusion membrane, suggesting an additional role for PmpD in maintaining RB-inclusion interactions (Kari et al., 2014). Infection in non-human primates with these PmpD null mutants showed decreased chlamydial burden illustrating its role in pathogenesis (Kari et al., 2014). Although all Pmps are involved in adhesion, it remains unclear what the relative importance of each Pmp to CT is. *pmpA*, *pmpD* and *pmpI* are the most conserved CT Pmp genes and their expression are also unaffected by penicillin-induced stress, suggesting a greater selection pressure to maintain those genes relative to other Pmps (Carrasco et al., 2011).

Pmps may also play an important role in modulating the immune response. In *C. pneumoniae* and *C. psittaci*, the N-terminal Pmp21/PmpD activates Toll-like receptor 2 (TLR2), myeloid differentiation factor 88 (MYD88) and nuclear factor κB (NF-κB) signalling leading to Th2 polarised macrophages and upregulation of various cytokines/chemokines including IL-8, IL-6, IL-10 and monocyte chemoattractant protein-1 (MCP-1) (Wehrl et al., 2004). Importantly, Th2 polarised macrophages were associated with reduced nitric oxide (NO) production and *Chlamydia* killing (Chu et al., 2020). Whether CT Pmps also can modulate the host immune response remains to be seen.

Finally, genomic evidence has suggested that Pmps may be associated with tissue tropism and adaptation. Phylogenetic typing using 6 *pmp* genes (*pmpB*, *pmpC*, *pmpF*, *pmpG*, *pmpH* and *pmpI*) were able to separate CT strains into their respective biovars and serotypes (Gomes et al., 2006) These genes were under positive selection in one or more niches (Borges et al., 2012), suggesting that Pmps are involved in driving specific host-cell interactions or disease outcomes. However, the mechanisms of how these unique Pmp mutations contribute to phenotypic or virulence differences in CT are yet to be elucidated and are likely to benefit from fluorescence-reported allelic exchange mutagenesis (FRAEM) approaches now available (Mueller et al., 2016).

## 5 Cytotoxin

The human CT urogenital serovars (D-K) encode a partial 73 kDa cytotoxin (CT166) (Carlson et al., 2004). This cytotoxin was found to contain a functional glycosyltransferase DXD domain and UDP-glucose binding domain with significant homology to the large cytotoxins (LCT) from *Clostridioides difficile (* Belland et al., 2001 *)*. The chlamydial cytotoxin is responsible for the cytopathic ballooning effects seen in infected host cells by glucosylating the Rho-GTPase protein, Rac1. This inactivates Rac1 leading to actin remodelling (Thalmann et al., 2010). In addition to Rac1, the cytotoxin also glucosylates other small GTPases including H-Ras, K-Ras and N-Ras. This was found to inhibit ERK1/2 and P13K/Akt signalling pathways leading to reduced cell cycle progression, division, and migration (Bothe et al., 2015). Crucial to Rac1 and Ras glucosylation is the DXD motif with mutations in this motif shown to abrogate glucosylation (Thalmann et al., 2010; Bothe et al., 2015). However, DXD mutated cytotoxin was still capable of reducing cellular migration suggesting that there may be other toxin effects independent to the DXD motif (Bothe et al., 2015).

Currently, the importance and role of the cytotoxin in CT pathogenesis is unclear. CT166 is present in EBs at the start of infection but is rapidly degraded after 2 hours post-infection (Belland et al., 2001). This suggests potential importance in the initial stages of infection. Ectopic overexpression of CT166 in HeLa cells reduced CT uptake. It was suggested that the chlamydial cytotoxin controls *Chlamydia* uptake and functions to limit excessive actin polymerisation from other virulence factors such as Tarp (Thalmann et al., 2010). The cytotoxin has also been hypothesised to preserve host energy and nutrients for *Chlamydia* by limiting energy intensive processes such as cell division and migration (Bothe et al., 2015).

Only urogenital serovars encode a functional cytotoxin. In ocular serovars, the glycosyltransferase domain is deleted while in LGV serovars, both domains are absent (Carlson et al., 2004). It has been argued that the progressive loss of the cytotoxin gene in CT compared to other *Chlamydia* species like *C. muridarum*, which encodes 3 paralogous copies, may reflect host adaptation and that the cytotoxin is redundant for human infection.

## 6 Plasmid

*Chlamydia* retains a highly conserved ~7.5 kb virulence plasmid that was found to be significant *in vivo* (Zhong, 2016). The plasmid encodes 8 glycoproteins (pGP1-8) that have a variety of functions including promoting infection ascension, inducing pro-inflammatory responses, and promoting extrusion processes. pGP4, is a putative transcriptional regulator of both plasmid and chromosomal genes (Turman et al., 2023). Virulence plasmid-deficient or pGP3-deficient strains resulted in attenuated infections (Carlson et al., 2008). Under stressful conditions, CT plasmid copy numbers increase, presumably to upregulate T3SS-secreted substrates and ensure *in vivo* survival (Dirks et al., 2021).

Expression of pGP3 is regulated by pGP4, which similarly is involved in regulating expression of chromosomal glycogen synthase (GlgA), and other proteins with currently unknown functions in pathogenesis, CT050, CT143, and CT144 (Lei et al., 2021). pGP4 was proposed to serve as a plasmid pGP4-dependent secretion system, imperative for delivering pGP3 and GlgA to the host cytosol to increase host glycogen stores (Lei et al., 2021). Contrary to previous results from (da Cunha et al., 2017) this secretion was independent of the T2SS and T3SS (Lei et al., 2021). pGP4-deficient CT strains demonstrate varied transcription of chromosomal and plasmid genes, responsible for various duties (e.g. glycogen synthesis and inclusion morphology) within the developmental cycle that impact virulence (Song et al., 2013). Additionally, pGP4 has been implicated in boosting Euo’s ability to bind and repress Euo-dependent transcription promoters (**Table 1A**), thus temporally altering late gene expression necessary for RB differentiation into infectious progeny (Zhang et al., 2020). However, plasmid-deficient CT is also capable of inducing sequelae as severe as wild-type strains containing the plasmid (Qu et al., 2015). Thus, the exact role of chlamydial plasmid’s function in pathogenesis remains unknown but can be investigated using new techniques such as FRAEM to create gene deletions (Mueller et al., 2016; Ghosh et al., 2020; Fields et al., 2022).

## 7 Other characterised chlamydial virulence factors

CT’s pathogenesis is additionally mediated by various proteases and membrane proteins. HtrA (DegP) is a periplasmic serine protease with chaperone functions, including proteolysis of abnormal or misfolded proteins, and serves as a stress response protein that is upregulated in some persistence and stress models (e.g. high temperatures, antibiotics) (Huston et al., 2007; Huston et al., 2008). Little was known about the exact role HtrA played in *Chlamydia*’s pathogenesis prior to inhibition studies with JO146 (Patel et al., 2014). This inhibitor was most effective in CT’s mid-replication cycle, where administration of the drug resulted in a significant loss in overall vacuole size and in viable infectious EB progeny (Gloeckl et al., 2013; Ong et al., 2013; Lawrence et al., 2016).

Chlamydia Protease-like Activity Factor (CPAF) is a secreted protease that was initially thought to degrade dozens of host proteins involved in Golgi reorganisation, apoptosis, cytoskeleton remodelling and immune regulation. However, these initial CPAF targets identified were disproven as artefacts from *in vitro* post-lysis degradation, as CPAF is not inhibited by standard protease inhibitors (Chen et al., 2012). To clarify the role of CPAF, a reverse genetics approach re-established CPAF’s likely functions in targeting specific host cell proteins (i.e. vimentin and lamin-associated protein 1) impacting on late stages of infection and development of infectious progeny, however CPAF is not essential for either process (Snavely et al., 2014). Additional experiments also revealed CPAF as a key factor for evading host immune response by paralysing early recruitment of polymorphonuclear cells. Secreted CPAF cleaves FPR2 on the surface of neutrophils, inhibiting their activation, oxidative bursts and production of neutrophil extracellular traps thus establishing longer infections (Rajeeve et al., 2018).

Other important CT proteases include Ptr which is localised to the inclusion lumen and is expressed throughout CT’s development. A CT LGV-L2 *ptr*-null mutant demonstrated impaired genome replication after IFN-γ removal, along with decreased progeny generation (Panzetta et al., 2019). This links Ptr’s role in recovering from IFN-γ-induced persistence. The periplasmic Tail-specific protease, Tsp, has also been identified as critical for the differentiation from RB to EB via genetic approaches which alter Tsp expression levels (Swoboda et al., 2023).

Lastly, the major outer membrane protein (MOMP) is an immunodominant antigen. It functions as a porin, a cytoadhesin, and is the dominant component of the outer membrane complex that maintains EB stability (Wang et al., 2006; Guifeng et al., 2007).

## 8 Discussion and conclusion

Genetic approaches to investigate CT are still time consuming and technically difficult. Genome wide genetic manipulation and screening approaches remain challenging. Therefore, selective approaches to genetic manipulation guided by current knowledge of virulence functions, homolog functions, etc. has been used to target loci for genetic manipulation. These genetic approaches in CT have already confirmed the role of several established virulence factors while also overturning or disputing other widely-held understandings in *Chlamydia* pathogenesis such as the role of the T3SS in secreting plasmid regulated chromosomally-encoded proteins and the role of CPAF (Snavely et al., 2014; Lei et al., 2021). While significant advances in genetic approaches have been made in CT, the progress in other *Chlamydia* species remains behind. Application or modification of genetic approaches in CT like CRISPRi to other species will allow comparative studies to reveal important determinants of host tropism. Moreover, the recent development of a multiplexed CRISPRi knockdown system in CT is an exciting advancement which will allow interactions between two or more genes to be investigated, providing a more wholistic systems understanding of chlamydia pathogenesis (Hatch and Ouellette, 2023). Ultimately, rapidly expanding the arsenal of genetically investigated virulence factors in CT could inform novel treatment approaches or future attempts to develop a live attenuated vaccine strain.

## Author contributions

BJ: Visualization, Writing – original draft, Writing – review & editing. CF: Supervision, Writing – review & editing. WH: Conceptualization, Supervision, Writing – review & editing. LL: Conceptualization, Supervision, Writing – original draft, Writing – review & editing.
