# Corrigendum: Molecular pathogenesis of *Chlamydia trachomatis*

**DOI:** 10.3389/fcimb.2023.1358553

**Published:** 2024-01-05

**Authors:** Brittany Jury, Charlotte Fleming, Wilhelmina M. Huston, Laurence Don Wai Luu

**Affiliations:** ^1^ School of Life Sciences, Faculty of Science, University of Technology Sydney, Ultimo, NSW, Australia; ^2^ Faculty of Science, University of Technology Sydney, Ultimo, NSW, Australia

**Keywords:** *Chlamydia trachomatis*, pathogenesis, virulence, immune evasion, type III secretion system, proteases, cytotoxin, polymorphic membrane proteins

In the published article, there were errors in some sections of the text where we referred to the wrong gene, citation or did not clearly articulate previous findings. These are detailed below:

A correction has been made to **Section 2 Transcription and regulatory factors as a target to understand pathogenesis, paragraph 3** where we said phase-locked σ^54^ mutants were used instead of CtcC mutants to investigate the transcriptional profiles for the σ^54^ regulon.

This sentence previously stated:

*“Recently, transcriptional profiles for the σ^54^ regulon was generated using a phase-locked σ^54^ mutant which further revealed the role of this sigma factor in membrane remodelling and incorporating T3SS effectors into EBs during RB differentiation into infectious EB progeny (Soules et al., 2020).”*

The correct sentence appears below:

*“Recently, transcriptional profiles for the σ^54^ regulon was generated using CtcC (an ATP-hydrolyzing response regulator for σ^54^) mutants which further revealed the role of this sigma factor in membrane remodelling and incorporating T3SS effectors into EBs during RB differentiation into infectious EB progeny (Soules et al., 2020).”*

A correction has been made to **Section 3.3 Inc proteins, paragraph 1 and 3** where we erroneously referred to IncS instead of CTL0390 and we have provided further clarity on previous findings on Inc proteins.

This section previously stated:

*“Inc proteins, or inclusion membrane proteins, are characterised by the presence of SNARE-like motifs in their coding sequence and have a diversity of functions. “…”IncA is required for inclusion vacuole fusion* (Table 1B)*, other functions of Inc proteins include; host cell viability (IncG), establishing infection (IncD) and promoting replication (IncV).” …”Here, we focus on recent IncM, IncS and CpoS which have been recently characterised.”*

*“The IncS effector is important for mediating EB exit via host-cell lysis during the late stages. IncS regulates Golgi translocation and activation of STING, which is needed for host-cell lysis and bacterial exit at the correct development stage for typical growth (Bishop and Derré, 2022).”*

The corrected section appears below:

*“Inc proteins or inclusion membrane proteins, share bilobed hydrophobic domains enabling anchoring to the inclusion membrane. Some Incs contain the presence of vesicle-targeting SNARE-like motifs in their coding sequence (e.g. IncA), which may facilitate host-pathogen interactions (Cingolani et al., 2019; Paumet et al., 2009).”…”IncA is required for inclusion vacuole fusion* (**Table 1B** )*, other functions of Inc proteins include; a possible role in maintaining host cell viability (IncG) (Scidmore and Hackstadt, 2001; Verbeke et al., 2006) and as an early Inc, IncD may be important for establishing the inclusion as a replicative niche (Shaw et al., 2000), although further studies are required to confirm this.”…”Here, we focus on IncM, CTL0390 and CpoS which have been recently characterised.”*

*“The CTL0390 Inc effector is thought to play a role in mediating EB exit via host-cell lysis during the late stages. A CTL0390 mutant was observed to have reduced Golgi translocation of STING, which is likely required to regulate host-cell lysis for bacterial exit (Bishop and Derré, 2022).”*

A correction has been made to **Section 3.4 ChlaDUB, paragraph 1** where the sentence erroneously stated that ChlaDUB1 has been shown to stabilize GLUT-1 and GLUT-3, but the referenced study only showed ChlaDUB1 stabilizes GLUT-1.

This sentence previously stated:

*“ChlaDUB1 also prevents degradation of host glucose-transporter-1 (GLUT-1) and GLUT-3 proteins (Wang et al., 2017). This likely supports growth as knockdown of host GLUT-1, GLUT-3 and adolase A impairs C. trachomatis infection (Wang et al., 2017).”*

The corrected sentence appears below:

*“ChlaDUB1 also prevents degradation of host glucose-transporter-1 (GLUT-1) proteins (Wang et al., 2017). This likely supports growth as knockdown of host GLUT-1 impairs C. trachomatis infection (Wang et al., 2017).”*

A correction has also been made to **Section 6 Plasmid, paragraph 1** where the sentence erroneously stated pGP3 as a transcriptional regulator instead of pGP4.

This sentence previously stated:

*“pGP3, is a master transcriptional regulator of both plasmid and chromosomal genes (Turman et al., 2023).”*

The corrected sentence appears below:

*“pGP4, is a putative transcriptional regulator of both plasmid and chromosomal genes (Turman et al., 2023).”*

The authors apologize for these errors and state that this does not change the scientific conclusions of the article in any way. The original article has been updated.

